# Multimodality Imaging of LEOPARD Syndrome: Myocardial Hypertrophy With Diffuse Coronary Artery Dilation

**DOI:** 10.1016/j.jaccas.2022.07.002

**Published:** 2022-08-17

**Authors:** Dan Yang, Yining Wang, Minjie Lu

**Affiliations:** a Department of Magnetic Resonance Imaging, Fuwai Hospital, State Key Laboratory of Cardiovascular Disease, National Center for Cardiovascular Diseases, Chinese Academy of Medical Sciences and Peking Union Medical College, China; b Department of Magnetic Resonance Imaging, Xinyang Central Hospital, Henan, China; c Key Laboratory of Cardiovascular Imaging (Cultivation), Chinese Academy of Medical Sciences, Beijing, China

**Keywords:** cardiac magnetic resonance, differential diagnosis, follow-up, hypertrophic cardiomyopathy, LEOPARD syndrome, multimodality imaging, CMR, cardiac magnetic resonance, HCM, hypertrophic cardiomyopathy, LS, LEOPARD syndrome, LVOT, left ventricular outflow tract, TTE, transthoracic echocardiogram

## Abstract

We describe the case of a 5-year-old girl with genetically confirmed LEOPARD syndrome (LS) who presented with multiple lentigines, ocular hypertelorism, retardation of growth, myocardial hypertrophy, and diffuse coronary artery dilatation. This case highlights the importance of multimodality imaging for the assessment of LS-associated cardiovascular alterations and follow-up. (**Level of Difficulty: Intermediate.**)

## Central Illustration

**Figure:**
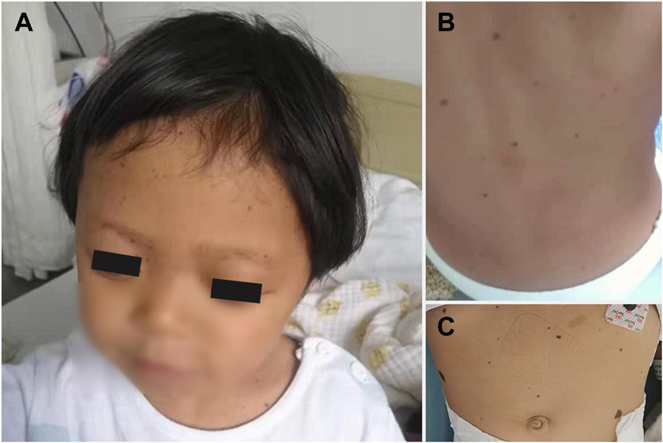

A 5-year-old girl was admitted to our hospital with suspected recurrent obstruction of the left ventricular outflow tract (LVOT) by physical examination 4 years after surgery for hypertrophic cardiomyopathy (HCM).

**Learning Objectives**

• To understand the conception and differential diagnosis of LS.
• To understand the role of multimodality imaging in the diagnosis of LS.
• To recognize the necessity and importance of imaging follow-up for assessment of LS- associated cardiovascular alterations.

**Figure 1:**
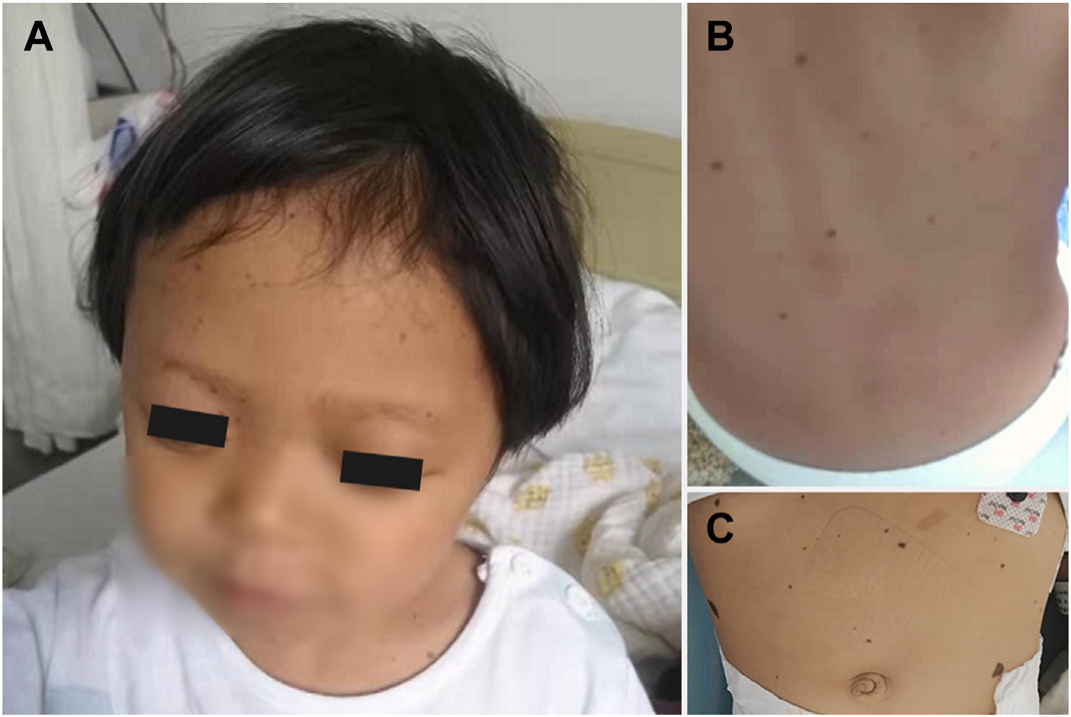
Multiple Lentigines and Dysmorphic Features **(A)** Hypertelorism. **(A to C)** Diffuse lentigines on the forehead, eyebrow, neck, back, and abdomen.

She was afebrile, with a heart rate of 120 beats/min, blood pressure of 102/56 mm Hg, and respiratory rate of 30 breaths/min. Physical examination revealed multiple lentigines scattered on the skin, ocular hypertelorism (Figure 1), short stature, a language disorder without deafness, abnormal genitalia, and other dysmorphic features. Cardiac examination revealed systolic murmurs that were heard along the left sternal border in the second intercostal space and the right sternal border in the fourth intercostal space.

## Medical History

The child had been found to have a heart murmur at birth. Her transthoracic echocardiogram (TTE) showed myocardial hypertrophy with biventricular outflow tract obstruction and mild coronary artery dilatation with a diameter of 2.3 mm for the right coronary artery and 3.6 mm for the left coronary artery. At 1 year of age, she underwent surgery in a local hospital to reduce the outflow tract obstruction by resection of the biventricular anomalous muscle bundles. Her family history showed that her paternal uncle and aunt had a history of myocardial hypertrophy without clinical symptoms.

## Differential Diagnosis

The differential diagnosis for this disorder mainly included other RASopathies, a group of developmental disorders caused by mutations in genes that encode regulators of the RAS/MAPK pathway.^1^ They share common clinical features, such as distinctive facial features, cardiopathies, growth and skeletal abnormalities, and mental retardation, including Noonan syndrome, cardiofaciocutaneous syndrome, and neurofibromatosis type I.

## Investigations

**Figure 2:**
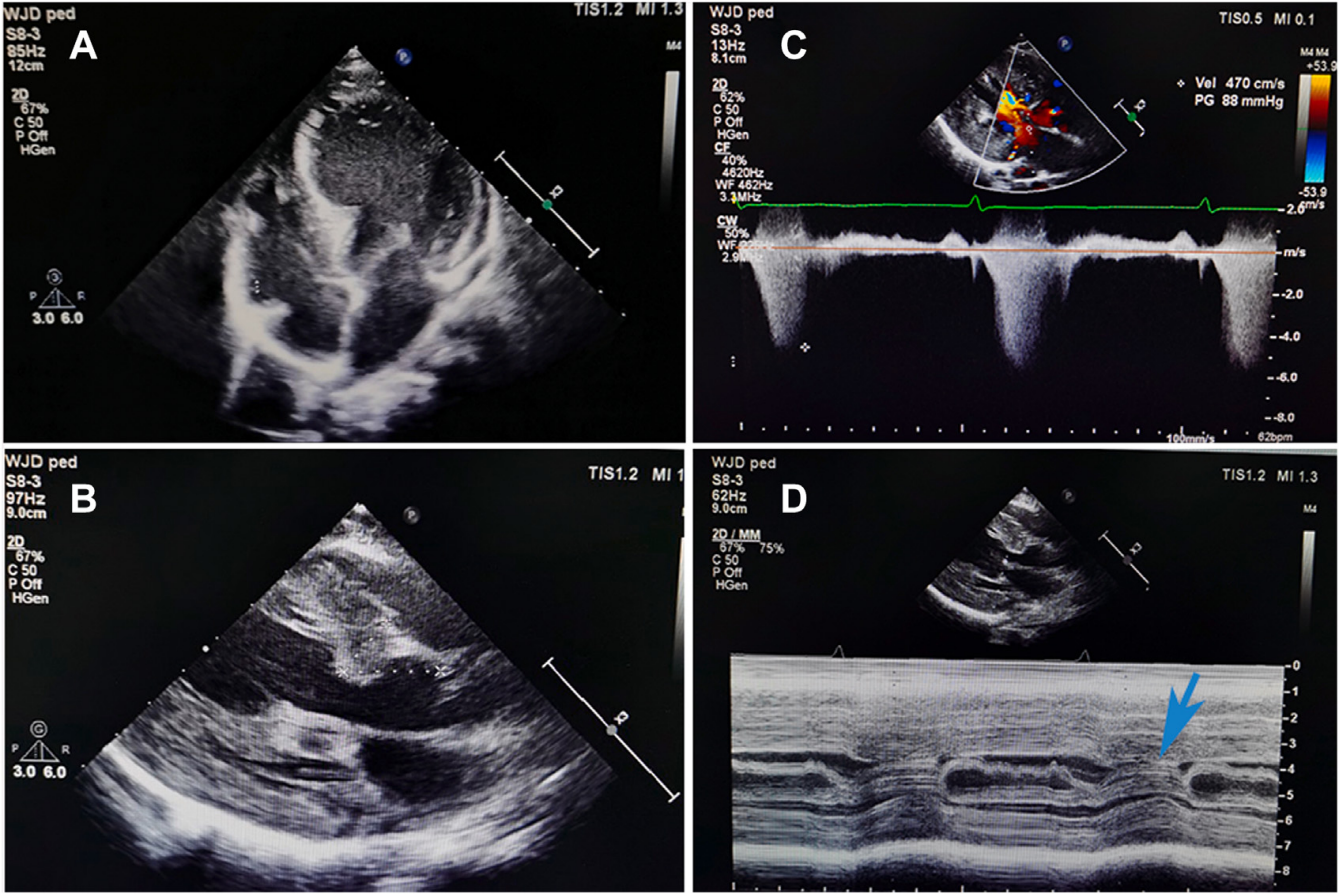
Transthoracic Echocardiogram **(A)** Four-chamber view at end diastole and **(B)** parasternal long-axis view at end systole show anatomical findings of increased wall thickness. **(C)** Continuous-wave Doppler **(C)** reveals a maximum velocity of 4.7 m/s and a calculated peak gradient of 88 mm Hg through the left ventricular outflow tract (LVOT) at rest. **(D)** M-mode echocardiogram shows systolic anterior motion of the mitral valve **(blue arrow)**, indicating LVOT obstruction.

A TTE revealed asymmetric myocardial hypertrophy, mild left atrial enlargement, and systolic anterior motion of the mitral valve with moderate regurgitation. Continuous-wave Doppler demonstrated a calculated peak gradient of 88 mm Hg (maximum velocity of 4.7 m/s) across the LVOT at rest, indicating severe LVOT obstruction (Figure 2, Videos 1 and 2).

**Figure 3:**
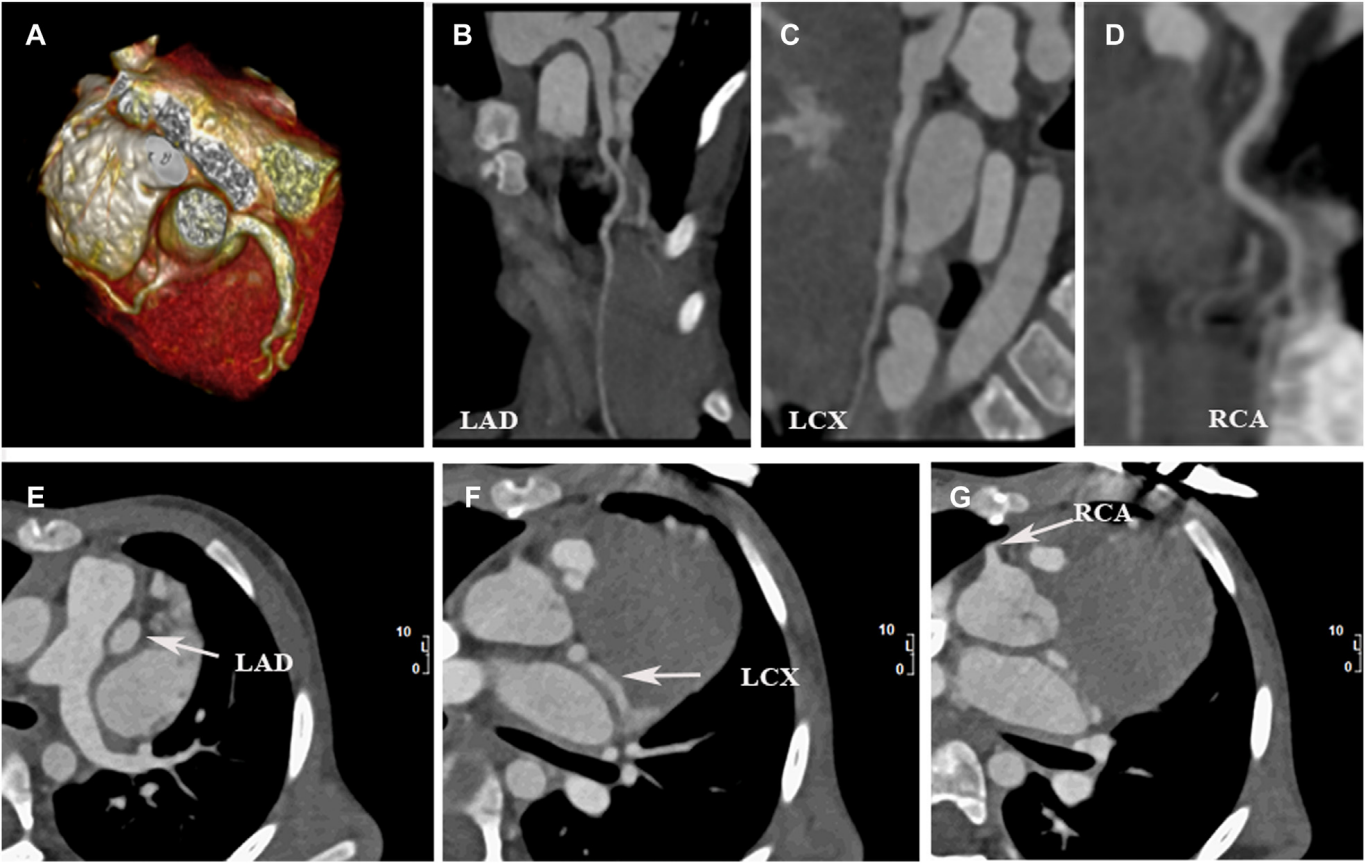
Coronary Computed Tomography Angiography **(A)** Three-dimensional volume-rendered image shows normal origins of coronary arteries. **(B to D)** Curved planner reformation demonstrates that coronary artery lumen is unobstructed. **(E to G)** Axial images show diffuse dilatation of coronary arteries **(white arrow)**, with a maximum diameter of 3.6 mm for the right coronary artery (RCA) and 5.3 mm for the left anterior descending (LAD) artery. LCX = left circumflex artery.

Coronary computed tomography angiography showed diffuse dilatation of the coronary arteries; especially the left coronary artery, without thrombus formation in the lumen. There was no coronary arterial fistula or other coronary malformation (Figure 3).

**Figure 4:**
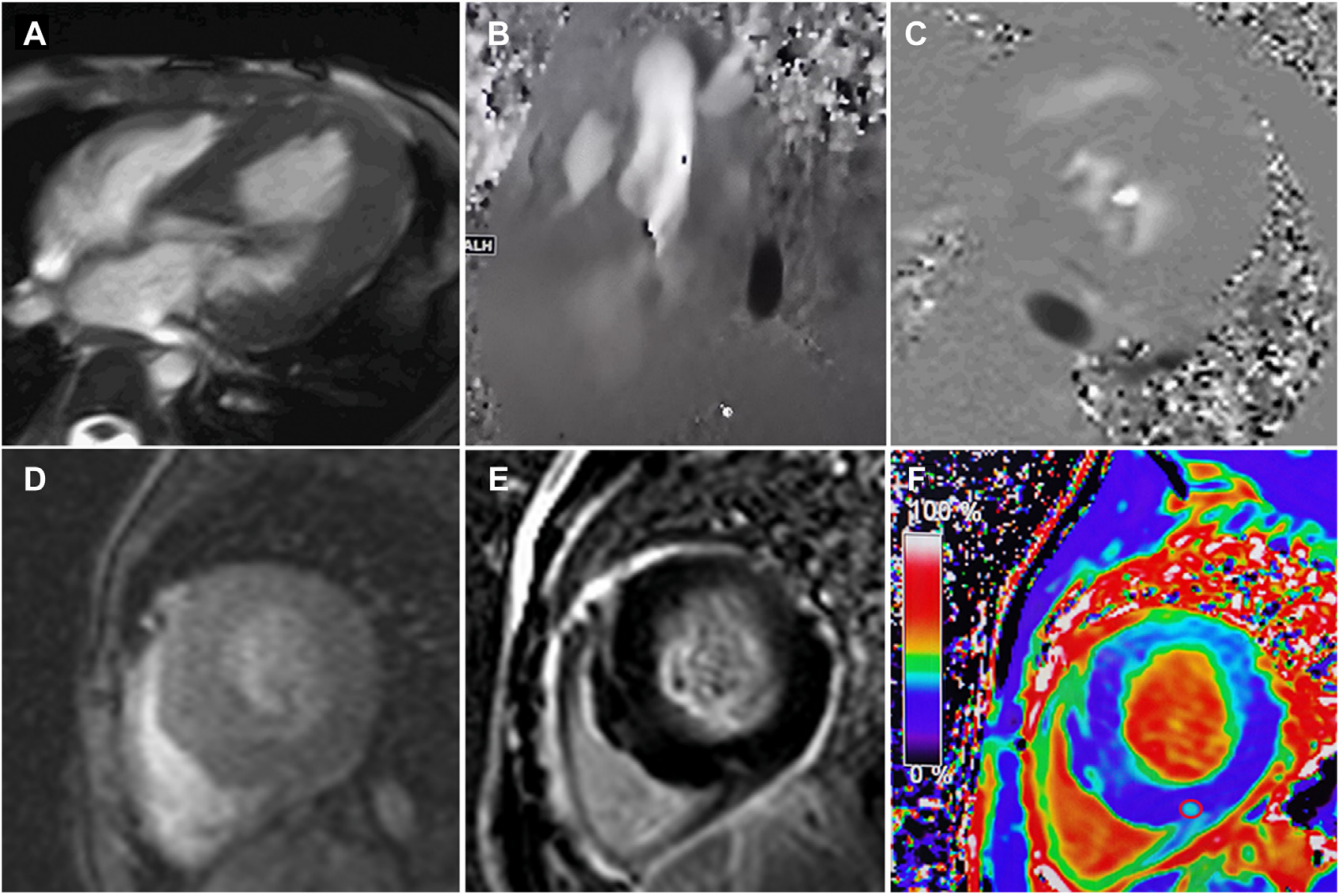
Cardiac Magnetic Resonance **(A)** Cine magnetic resonance, 4-chamber view at end diastole shows diffuse myocardial hypertrophy, especially basal septum. **(B, C)** Phase-contrast flow and velocity imaging shows flow acceleration through the narrowed left ventricular outflow tract during contraction of the left ventricle. **(D)** Myocardial perfusion imaging is normal. **(E)** Late enhancement on phase-sensitive inversion recovery image demonstrates a patch of late gadolinium enhancement at the right ventricular insertion site, where the extracellular volume value **(red circle)** is increased (about 39%) (**F**).

Cardiac magnetic resonance (CMR) showed diffuse myocardial hypertrophy, particularly in the basal interventricular septum, with a masslike protrusion toward the LVOT. This led to severe LVOT obstruction where flow acceleration and systolic anterior motion of the mitral valve were present. The left ventricular systolic function was mildly decreased, with a left ventricular ejection fraction of 52%; all of the above were similar to the results of the TTE. A patch of late gadolinium enhancement was observed in the interventricular septum, which indicated less myocardial fibrosis, an indicator for prognosis assessment. (Figure 4, Videos 3 and 4).

**Figure 5:**
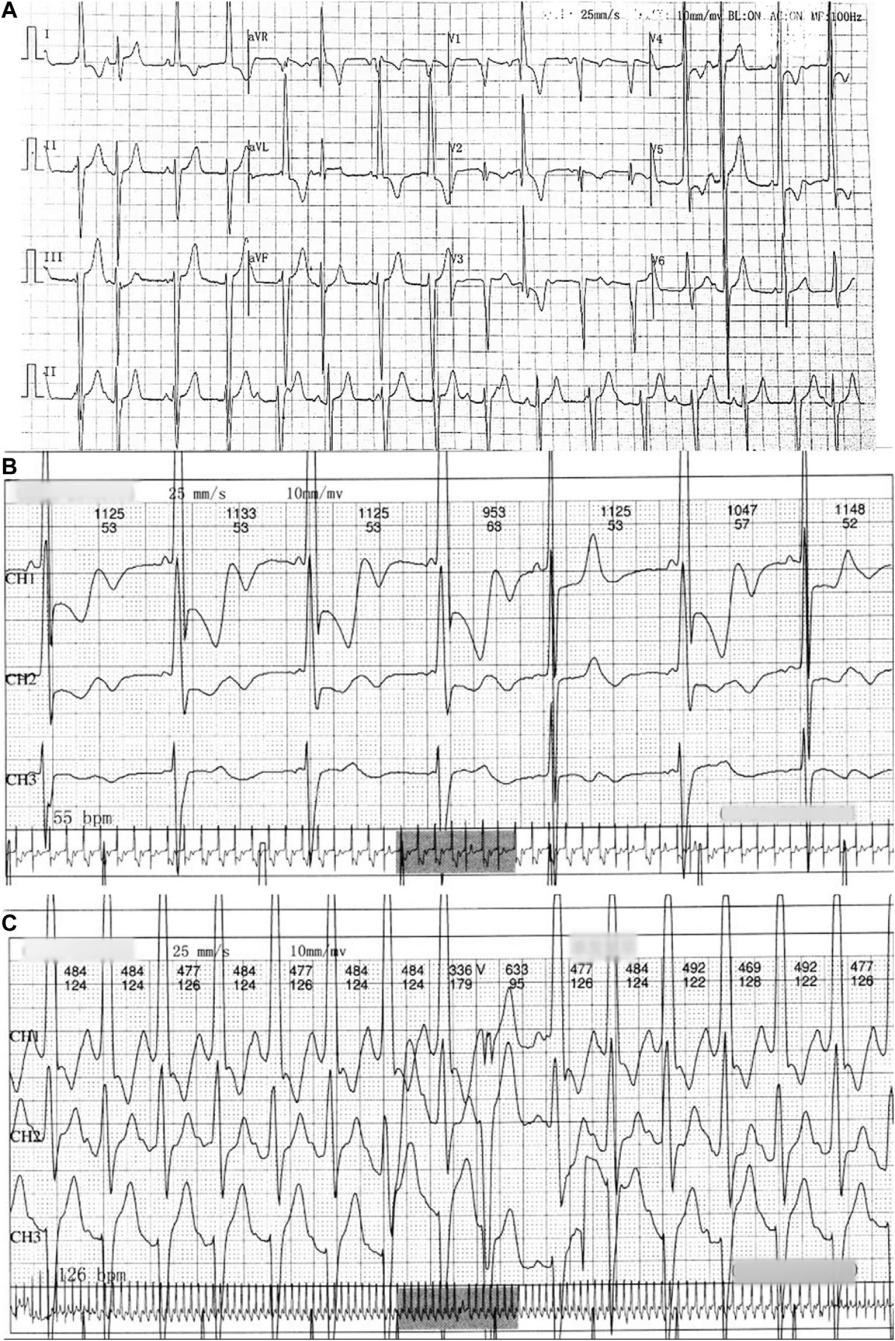
12-Lead Electrocardiogram and 24-Hour Holter Monitoring **(A)** Electrocardiogram shows sinus tachycardia, ventricular tachycardia with atrioventricular interference dissociation, incomplete right bundle branch block, and left ventricular hypertrophy, defined by SV1 + RV5 >3.5 mV. **(B)** Holter registration of a junctional escape rhythm. **(C)** Holter registration of a sinus tachycardia with aberrancy and a ventricular premature beat.

A 12-lead electrocardiogram showed sinus tachycardia, ventricular tachycardia with atrioventricular interference dissociation, incomplete right bundle branch block, and left ventricular hypertrophy. Twenty-four-hour Holter monitoring revealed a junctional escape rhythm, a sinus tachycardia with aberrancy, and a ventricular premature beat (Figure 5).

The results of laboratory examinations were unremarkable.

## Management

The patient underwent a second extended septal myectomy in our hospital. Subsequently, a TTE showed the LVOT gradient significantly decreased from 88 mm Hg to 19 mm Hg, and the cardiac murmurs disappeared. The patient recovered well and was discharged 13 days after undergoing cardiac surgery.

The patient underwent a genetic test, during which a heterozygous missense mutation (c.836A>G, p.Tyr279Cys) in exon 7 of the PTPN11 gene was detected. However, the genetic test results of her sisters were normal.

## Discussion

LEOPARD is an acronym for the initial letters of the characteristic symptoms: L, lentigines; E, electrocardiographic conduction abnormalities; O, ocular hypertelorism; P, pulmonary stenosis; A, abnormal genitalia; R, retardation of growth; and D, deafness (sensorineural).^2^ LEOPARD syndrome (LS) is a rare genetic disorder that affects multiple organs.

LS is a phenotypic expression of an allelic disorder mainly caused by different missense mutations in PTPN11, a gene encoding the protein tyrosine phosphatase SHP-2 located at band 12q24.1, which regulates intracellular signaling and controls several distinct developmental processes.^3^ Two common mutations of LS are located in exons 7 (p.Tyr279Cys) and 12 (p.Thr468Met).^4^ In genotype-phenotype correlation, the mutation Y279C is more frequently associated with short stature, deafness, and myocardial hypertrophy.^5^ According to Voron et al,^6^ in the presence of multiple lentigines, the clinical diagnosis of LS may be suspected if there are at least 2 cardinal features. In our case, the patient demonstrated the typical phenotype of hypertrophic obstructive cardiomyopathy with diffuse coronary artery dilation, an uncommon manifestation of LS.^7^^,^^8^ Simultaneously, she had multiple lentigines, a facial deformity, retardation of growth, and electrocardiographic abnormalities. On the basis of the above pathognomonic criteria, the diagnosis of LEOPARD syndrome was confirmed.

The role of imaging in the diagnosis of this rare disease is mainly to assess cardiac involvement. The TTE could be a routine first-line imaging modality to evaluate the morphology and function of cardiac involvement. CMR is an alternative modality to more accurately assess cardiac structure and function, and further evaluation of myocardial tissue characterization can be performed by late gadolinium enhancement and/or T1 mapping and extracellular volume. Coronary computed tomography angiography is also recommended, especially when the TTE or CMR suggests coronary artery involvement.

Not only is HCM particularly common in LS, but LS-associated HCM presents at the most severe end of the phenotypic spectrum.^9^ Myocardial hypertrophy in LS is indeed associated with a risk of fatal cardiac events, as seen in primary HCM.^4^ Thus, regular follow-up imaging is necessary to avoid adverse cardiovascular events. Furthermore, screening for coronary artery abnormalities is crucial because of the risk of thrombus formation, which can develop in dilated coronary arteries.^7^

## Follow-Up

The patient recovered well from surgery and was treated with β-blockers and diuretics after discharge.

## Conclusions

Early-onset diffuse left ventricular hypertrophy with abnormal appearance and other organ dysfunction requires suspicion of HCM with nonsarcomeric mutations. Multimodality imaging is crucial for obtaining a diagnosis and eliminating differential diagnoses, and also for perioperative management and prognostic prediction for these rare genetic diseases.

## Funding Support and Author Disclosures

This work was supported by the Construction Research Project of the Key Laboratory (Cultivation) of Chinese Academy of Medical Sciences (2019PT310025), National Natural Science Foundation of China (81971588), Youth Key Program of High-level Hospital Clinical Research (2022-GSP-QZ-5). The authors have reported that they have no relationships relevant to the contents of this paper to disclose.

## Appendix

Supplemental Video 1 Transthoracic Echocardiogram. Apical 4-chamber view shows asymmetric hypertrophy of the LV with the septum concave to the LV cavity and a prominent basal septal bulge.

Supplemental Video 2 Transthoracic Echocardiogram. Parasternal long-axis view using color Doppler depicts the left ventricular outflow tract narrowness with the high-velocity flow and moderate mitral regurgitation.

Supplemental Video 3 Cardiac Magnetic Resonance. Cine CMR, short-axis views showing asymmetric left ventricular hypertrophy and mild systolic dysfunction. S

Supplemental Video 4 Cardiac Magnetic Resonance. Cine CMR, 3-chamber view showing end-systolic left ventricular outflow tract narrowing and systolic anterior motion of the mitral valve.
